# Neurocognitive and Emotional Outcomes in Childhood Cancer: A Developmental Perspective

**DOI:** 10.3390/curroncol32110611

**Published:** 2025-11-01

**Authors:** Antonios I. Christou, Georgia Kalfadeli, Stella Tsermentseli, Flora Bacopoulou

**Affiliations:** 1 Department of Special Education, University of Thessaly, 38221 Volos, Greece; gkalfadeli@uth.gr; 2 Department of Primary Education, University of Thessaly, 38221 Volos, Greece; tsermentseli@uth.gr; 3 Clinic for Assessment of Adolescent Learning Difficulties, Center for Adolescent Medicine and UNESCO Chair in Adolescent Health Care, First Department of Pediatrics, Medical School, National and Kapodistrian University of Athens, 11527 Athens, Greece; fbacopoulou@med.uoa.gr

**Keywords:** childhood cancer survivors, executive functions, emotional development, neuropsychological outcomes, emotional regulation, late effects, anticancer treatment

## Abstract

Background: Childhood cancer survivors (CCSs) are at heightened risk of long-term neurocognitive and emotional difficulties that can affect educational attainment, social participation, and overall quality of life. These outcomes vary across developmental stages and are influenced by treatment modality, age at diagnosis, and central nervous system (CNS) involvement. Methods: A comprehensive literature search was conducted in PubMed, Scopus, PsycINFO, and Web of Science for articles published between January 2000 and June 2024. Search terms included combinations of “childhood cancer survivors,” “neurocognitive outcomes,” “executive function,” “emotional regulation,” and related MeSH terms. Inclusion criteria required peer-reviewed studies assessing CCS using standardized neuropsychological or emotional measures. Results: Evidence indicates persistent deficits in processing speed, working memory, and higher-order executive functions, with additional challenges in attention and memory. Emotional difficulties, including anxiety, depression, and social withdrawal, were prevalent and often co-occurred with cognitive impairments. Developmental timing of cancer and treatment was a key determinant of outcome. Family functioning, school reintegration support, and broader social environments emerged as important moderators of resilience. Conclusions: CCSs face complex, interrelated cognitive and emotional challenges that warrant early identification and ongoing, developmentally tailored intervention. Integrated approaches combining cognitive remediation and psychosocial support appear most effective. Future research should prioritize longitudinal designs, multi-informant assessments, and culturally sensitive frameworks to inform targeted prevention and rehabilitation strategies. Our synthesis highlights that deficits in processing speed and working memory are most pronounced following CNS-directed therapies during early developmental stages, whereas emotional vulnerabilities such as anxiety and social withdrawal often emerge later in adolescence. Interventions combining cognitive remediation, targeted psychosocial support, and structured school reintegration show the strongest evidence for improving adaptive outcomes. Coordinated survivorship care across healthcare, educational, and family systems is essential to sustain developmental recovery.

## Simple Summary

Many children survive cancer, but some continue to face challenges in thinking skills and emotions for years after treatment. These may include problems with memory, attention, and planning, as well as feelings of anxiety, sadness, or difficulty connecting with others. Our review looked at recent research on how these difficulties appear at different ages, from early childhood to adolescence, and what factors influence recovery. We found that the type of treatment, age when cancer was diagnosed, and support from family and school all make a difference. The good news is that combining brain training with emotional and social support can help children and teenagers do better over time. Understanding these challenges, and ensuring coordination between families, teachers, and healthcare professionals, can guide the provision of the right help at the right stage of development.

## 1. Introduction

Childhood cancer survival rates have improved dramatically over the past decades, largely due to advances in diagnostic techniques, treatment protocols, and supportive care [1,2]. Current estimates indicate that over 80% of children diagnosed with cancer now survive at least five years post-diagnosis, with many living into adulthood [3,4]. This success has shifted the focus of pediatric oncology from survival alone to the quality of life and long-term functional outcomes for survivors [5]. As the population of childhood cancer survivors (CCSs) grows, it has become increasingly important to address the late effects of treatment, which may persist for years after remission and can influence educational attainment, social integration, and emotional well-being [6,7].

Among the most prevalent late effects are neurocognitive and emotional difficulties, which may emerge months or even years following treatment completion [8,9]. These impairments can affect multiple domains, including processing speed, attention, memory, executive function, emotional regulation, and social functioning [10,11,12]. While some survivors experience gradual improvement, others may show stable or progressive deficits, underscoring the need for ongoing monitoring and intervention [13].

The causes of these late effects are multifactorial, involving direct neurotoxic effects of cancer therapies, secondary medical complications, and environmental and psychosocial influences [14,15,16]. Treatments such as cranial irradiation, intrathecal chemotherapy, and high-dose systemic chemotherapy have been consistently linked to structural and functional changes in the developing brain, including white matter injury, cortical thinning, and altered connectivity in networks supporting cognitive and emotional processing [17,18,19,20]. Age at diagnosis, treatment intensity, and the presence of comorbidities such as hearing loss or endocrine dysfunction can further modulate risk [21,22].

The developmental stage at which treatment occurs plays a critical role in determining the nature and severity of late effects [23,24]. Brain maturation is a dynamic process, with different regions and networks developing at different times; thus, injury during sensitive periods can lead to specific, long-lasting impairments [25,26]. For example, younger children may be more vulnerable to broad and diffuse deficits, while older children and adolescents may show more focal impairments in executive function and psychosocial adaptation [27,28]. Understanding these developmental trajectories is essential for designing interventions that are both age-appropriate and context-sensitive.

In addition to biomedical factors, psychosocial and environmental contexts significantly influence survivor outcomes [29,30]. Supportive family environments, access to specialized educational services, and timely rehabilitation can buffer against the negative impacts of treatment-related neurocognitive decline [31,32,33]. Conversely, social isolation, school disengagement, and mental health difficulties can exacerbate cognitive and emotional challenges, creating a cycle of disadvantage [34,35]. Recent research highlights the bidirectional relationship between neurocognitive function and psychosocial well-being, suggesting that interventions should target both domains concurrently [36,37].

Several narrative and systematic reviews have examined late effects in CCSs, but most have either focused on a single domain (e.g., neurocognition or mental health) or lacked an explicit developmental framework [38,39,40]. Furthermore, earlier reviews often relied on studies conducted before the widespread adoption of contemporary treatment protocols, limiting their applicability to current survivor populations [41]. There is a need for an updated synthesis that integrates findings from recent research, considers developmental timing, and encompasses both neurocognitive and emotional domains.

Childhood cancer survivorship cannot be understood without a developmental lens. Each stage of growth brings unique neurocognitive and psychosocial tasks, from the acquisition of language in early childhood to identity formation in adolescence and independence in young adulthood. Cancer treatment intersects with these milestones in ways that can either derail or reshape developmental trajectories. This review therefore adopts a lifespan perspective, emphasizing how treatment timing, family context, and broader systems interact to influence survivors’ long-term outcomes. This review addresses critical gaps by providing a comprehensive, developmentally informed overview of neurocognitive and emotional outcomes in childhood cancer survivors (CCSs). We first situate late effects within a developmental framework, emphasizing how vulnerabilities and adaptive capacities shift across childhood, adolescence, and early adulthood. We then examine in detail the major neurocognitive and emotional domains affected by cancer and its treatments, with attention to their interplay with family dynamics and environmental influences. Methodological considerations in survivorship research are outlined to contextualize variability in findings and to guide future study design. We also review current practices in neuropsychological assessment, including the developmental appropriateness of tools and the factors that influence outcomes. Interventions and support strategies are considered across domains—cognitive rehabilitation, psychosocial and emotional support, school reintegration, and innovative therapies—highlighting how approaches can be tailored across developmental stages. The discussion integrates these strands to identify resilience and protective factors, clinical implications, and policy needs. Finally, we highlight priorities for future research and innovation, with the aim of informing multidisciplinary survivorship care strategies that optimize long-term developmental trajectories and quality of life for this growing population.

## 2. Methodological Approach

A comprehensive literature search was conducted to identify peer-reviewed studies examining neurocognitive and emotional outcomes in childhood cancer survivors (CCSs). Searches were performed in PubMed, Scopus, PsycINFO, and Web of Science from January 2000 to June 2024. The search strategy combined free-text keywords and Medical Subject Headings (MeSH) terms related to childhood cancer survivors, neurocognitive outcomes, executive function, emotional regulation, memory, and psychosocial outcomes.

To ensure transparency and reproducibility, the complete search strings for each database are provided below. Search syntax was adapted to the indexing conventions and Boolean operators of each platform.

PubMed:

(“childhood cancer survivors”[Title/Abstract] OR “pediatric oncology survivors”[Title/Abstract])

AND (“neurocognitive”[Title/Abstract] OR “cognitive”[Title/Abstract] OR “executive function”[Title/Abstract] OR “memory”[Title/Abstract] OR “attention”[Title/Abstract])

AND (“emotional regulation”[Title/Abstract] OR “psychosocial”[Title/Abstract] OR “mental health”[Title/Abstract])

AND (“2000/01/01”[Date—Publication]: “2024/06/30”[Date—Publication])

Scopus:

TITLE-ABS-KEY(“childhood cancer survivor*” OR “pediatric oncology survivor*”)

AND TITLE-ABS-KEY(“neurocognitive” OR “executive function” OR “memory” OR “attention”)

AND TITLE-ABS-KEY(“emotional regulation” OR “psychosocial” OR “mental health”)

AND (PUBYEAR > 1999 AND PUBYEAR < 2025)

PsycINFO (via APA PsycNet):

DE “Childhood Cancer” OR DE “Pediatric Oncology”

AND (DE “Neurocognitive Functioning” OR DE “Executive Function” OR DE “Memory” OR DE “Attention”)

AND (DE “Emotional Regulation” OR DE “Psychosocial Adjustment” OR DE “Mental Health”)

AND PEER(yes) AND Year ≥ 2000 AND Year ≤ 2024

Web of Science (Core Collection):

TS=(“childhood cancer survivor*” OR “pediatric oncology survivor*”)

AND TS=(“neurocognitive” OR “executive function” OR “memory” OR “attention”)

AND TS=(“emotional regulation” OR “psychosocial” OR “mental health”)

Refined by: DOCUMENT TYPES (Article OR Review) AND LANGUAGES (English)

Timespan: 2000–2024

For duplicate management, search outputs from all four databases were exported in RIS format and imported into EndNote 21. Automated duplicate detection was first performed using the “Find Duplicates” function, comparing title, author, and publication year fields. Remaining potential duplicates were verified manually. After deduplication, the unique record set was screened for eligibility according to the predefined inclusion and exclusion criteria.

To appraise methodological rigor, studies were evaluated using adapted criteria from the Joanna Briggs Institute checklist for observational studies. Each article was coded for sample size adequacy (>30 participants = low risk), presence of control/comparison groups, prospective versus retrospective design, and standardization of neurocognitive or emotional measures. Studies meeting ≥3 of 4 criteria were categorized as high quality, 2 as moderate, and ≤1 as low quality. Although no formal scoring was applied, this classification informed the interpretation of evidence across developmental stages.

The reference lists of relevant systematic reviews and meta-analyses were also hand-searched to identify additional eligible studies [1,2]. Studies were eligible for inclusion if they (a) involved participants diagnosed with cancer before 18 years of age, (b) reported on neurocognitive and/or emotional outcomes assessed using standardized measures, (c) included a minimum sample size of 15 participants to ensure statistical robustness, and (d) were peer-reviewed and published in English between January 2000 and June 2024. Exclusion criteria were as follows: (a) Studies focusing exclusively on adult-onset cancers, (b) case reports, editorials, conference abstracts, or dissertations without peer review, and (c) studies that assessed only physical or biomedical outcomes without cognitive or emotional components.

Given the heterogeneity in study designs, cancer types, treatment exposures, and outcome measures, a narrative synthesis approach was employed. Studies were grouped according to developmental stage at assessment: (a) Infancy and toddlerhood (0–2 years), (b) early childhood (3–5 years), (c) middle childhood (6–11 years), and (d) adolescence (12–18 years). Within each group, findings were further organized by cognitive domains (e.g., processing speed, working memory, and attention) and emotional/psychosocial outcomes (e.g., anxiety, depression, and social functioning).

Despite the breadth of evidence reviewed, methodological limitations remain. Considerable heterogeneity in cancer diagnoses, treatment exposures, and assessment tools constrains comparability across studies, while small sample sizes and cultural or linguistic biases may limit generalizability. These issues underscore the need for harmonized, longitudinal, and cross-cultural research designs.

## 3. Developmental Overview

Late effects in childhood cancer survivors (CCSs) must be understood within the context of normal brain and behavioral development, as the timing of treatment relative to developmental milestones can significantly shape outcomes [42,43]. Brain maturation follows a non-linear trajectory, with periods of rapid change—such as infancy, early childhood, and adolescence—representing windows of both opportunity and vulnerability [44,45].

During the first two years of life, rapid synaptogenesis, myelination, and sensory–motor integration lay the foundation for later cognitive, language, and socio-emotional skills [46,47]. Treatment-related insults at this stage—such as cranial irradiation, high-dose chemotherapy, or prolonged hospitalization—can disrupt these processes, often leading to delayed expressive language, impaired visual–motor integration, and early regulatory challenges (e.g., sleep or feeding difficulties) [48,49]. While some deficits may be apparent immediately, others may remain latent until school age, when task demands increase [50].

Early childhood (3–5 years) is characterized by a rapid expansion of vocabulary, emergence of basic executive functions (working memory and inhibitory control), and engagement in complex social play [51,52]. Neurotoxic treatments at this age can impair processing speed, attention, and social communication, with secondary effects on readiness for school [53]. Children treated during this stage may require early educational interventions, structured routines, and targeted support for language and play skills to prevent later cascading difficulties [54,55].

In middle childhood, cognitive demands increase substantially as formal schooling requires sustained attention, literacy and numeracy mastery, and task organization [56]. Treatment-related white matter injury and disrupted cortical connectivity can lead to processing speed deficits, working memory limitations, and academic underachievement [57,58]. These difficulties often become more pronounced over time, as academic demands exceed compensatory capacities [59]. Early neuropsychological assessment and individualized educational plans (IEPs) can mitigate long-term academic decline [60,61].

Adolescence involves refinement of higher-order executive functions, increased autonomy, and the development of complex social identities [62,63]. Treatments occurring during this period can interfere with prefrontal–limbic integration, resulting in challenges in planning, organization, emotional regulation, and social adaptation [64,65]. Survivors may face difficulties transitioning to higher education or employment, particularly if support services are withdrawn prematurely [66]. Interventions targeting self-management, mental health, and vocational readiness are critical at this stage [67].

**Figure 1:**
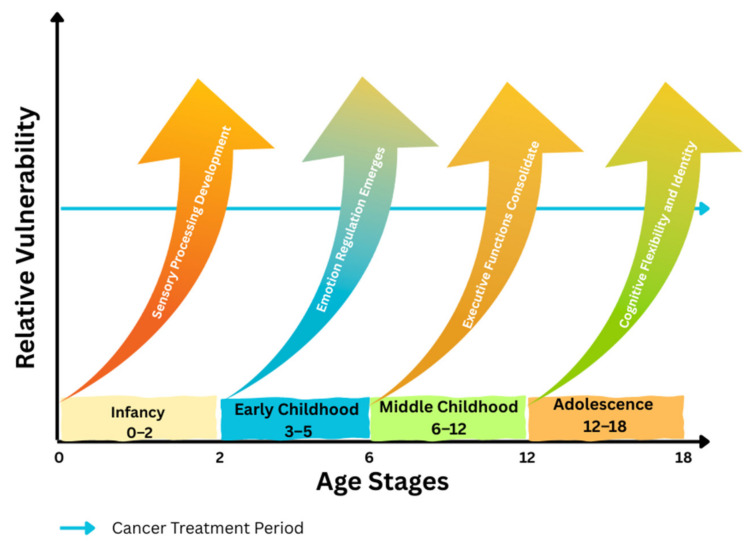
Timeline of Developmental Vulnerability: Key developmental stages and associated cognitive-emotional functions affected by cancer treatment.

The impact of treatment is moderated by a range of biological, psychosocial, and environmental factors. Younger age at diagnosis, higher treatment intensity, and co-existing medical complications (e.g., hearing loss, endocrine disorders) are associated with greater risk for both neurocognitive and emotional difficulties [68,69,70]. Protective factors include strong family support, access to specialized educational services, and timely psychosocial interventions [71,72]. Conversely, social isolation, stigma, and lack of coordinated survivorship care can exacerbate deficits [73,74]. To complement narrative synthesis, Figure 1 provides a conceptual model illustrating how treatment timing interacts with developmental stage to shape neurocognitive and emotional outcomes, moderated by family, school, and broader social environments.

Taken together, the evidence highlights that cancer and its treatment do not simply cause generic neurocognitive or emotional difficulties; rather, they alter the developmental trajectory at precisely those points when new skills or identities are emerging. Interventions at these sensitive periods can therefore have outsized effects, making developmental timing as critical a consideration as treatment exposure.

## 4. Neurocognitive Outcomes in Childhood Cancer Survivors

### 4.1. Infancy and Toddlerhood

During infancy and toddlerhood, rapid neural growth—including synaptogenesis, myelination, and the establishment of basic sensory–motor pathways—forms the foundation for later cognitive, language, and socio-emotional abilities [75,76]. At this stage, processing speed is expressed through rapid orienting to stimuli, quick sensory–motor responses, and early visual tracking. Cancer treatments involving cranial irradiation, high-dose methotrexate, or prolonged sedation in intensive care can slow neural conduction and disrupt white matter development, resulting in longer reaction times and less efficient integration of sensory information. These changes may not be apparent in simple daily routines but often become evident as the child encounters increasingly complex environmental demands in later years [77,78].

Attention and working memory are still in the earliest phases of development during this period, with attentional focus shifting quickly and the capacity for holding information in mind limited to only a few seconds. Disruptions due to repeated hospitalizations, fatigue, and reduced engagement with stimulating environments can restrict opportunities for attentional growth. For example, infants who spend extended periods in medical settings may have fewer chances to engage in exploratory play, joint attention activities, and caregiver–infant turn-taking, all of which are critical for developing early working memory skills [79].

Memory systems in this stage rely heavily on procedural and recognition memory, supported by subcortical structures, while declarative and episodic memory networks remain immature. Treatments that affect the hippocampus or disrupt early limbic system maturation can impair the encoding of new experiences, leading to subtler but significant consequences—such as reduced familiarity with caregiver routines or diminished novelty preference—that later translate into learning and recall difficulties in preschool and school-age years [80,81].

Early executive functions (EF)—such as inhibitory control, flexible shifting between activities, and early goal-directed behavior—are rudimentary in infancy. Treatment-related disruptions in prefrontal development can compromise these early EF capacities, though overt deficits may only emerge later when environmental demands for self-regulation increase. Nevertheless, subtle signs, such as difficulty soothing after stimulation or reduced persistence in play tasks, may signal early EF vulnerability [82].

The precursors of academic skills also begin to take shape in this period, including receptive and expressive language, symbolic representation in play, and early problem-solving through trial-and-error. Delays in these precursors—often linked to hearing loss from ototoxic chemotherapy or to prolonged sensory deprivation in hospital settings—can create a “developmental gap” that persists even after medical recovery [83].

Finally, motor and visual–motor integration undergo rapid maturation in the first two years. Neuropathy from certain chemotherapeutic agents, reduced muscle tone from prolonged inactivity, or disrupted cerebellar development can delay gross motor milestones such as sitting, crawling, and walking. Similarly, fine motor challenges—such as difficulty grasping and manipulating small objects—may affect later skills like handwriting and tool use [84]. Early intervention from physiotherapists and occupational therapists can mitigate some of these challenges, particularly when integrated into daily caregiving routines.

### 4.2. Early Childhood

Early childhood is marked by significant gains in language, social interaction, and the emergence of foundational academic skills. During this stage, processing speed becomes more apparent in the child’s ability to follow multi-step instructions, engage in back-and-forth conversations, and complete age-appropriate tasks within a reasonable timeframe. Cancer treatments involving CNS-directed chemotherapy or focal radiation can disrupt white matter maturation, slowing information transmission between brain regions [85,86]. This reduced processing efficiency may manifest as longer response latencies in conversation, difficulty keeping pace in group activities, or slower completion of pre-academic tasks such as sorting or matching.

Attention and working memory undergo rapid development at this age, enabling children to focus for longer periods and to hold and manipulate information mentally. However, survivors treated during this stage often show reduced attentional control and diminished working memory capacity, which may present as difficulty following multi-step instructions or sustaining focus during structured play [87]. Hospital-related absences from preschool and inconsistent participation in enrichment activities can further compound these challenges, leading to gaps in early learning readiness [88].

Memory abilities expand significantly in early childhood, with improvements in both short-term and long-term recall. Damage to hippocampal structures from neurotoxic agents can hinder the ability to encode and consolidate new information [89]. For example, children may have trouble remembering the sequence of a story or recalling newly learned vocabulary, which can slow language acquisition and narrative skills [90]. These deficits may become particularly evident in preschool assessments that require memory-based learning, such as recalling letter-sound associations or repeating patterns.

Executive functions such as inhibitory control, cognitive flexibility, and early planning skills begin to emerge more robustly during this period. Treatments that affect prefrontal cortex development may impair these capacities, resulting in impulsive responses, difficulty adapting to changes in routine, and struggles with transitioning between activities [91]. While such behaviors can be common at this age, persistent or exaggerated difficulties in survivors may signal underlying neurocognitive effects of treatment.

Academic precursors—including phonological awareness, counting skills, shape recognition, and pre-writing abilities—are actively developed in early childhood. Treatment-related neurocognitive deficits may interfere with the acquisition of these skills, especially in children with prolonged school absences or reduced access to enriched early learning environments [92]. Without targeted early intervention, these foundational gaps can evolve into persistent academic difficulties once formal schooling begins.

Motor and visual–motor skills also continue to refine in this stage, supporting activities such as drawing, cutting with scissors, and block building. Peripheral neuropathy, muscle weakness, or visual perception challenges secondary to treatment can delay mastery of these skills [93]. As fine motor skills are closely linked to early writing and self-care abilities (e.g., dressing and feeding), deficits in this area can have broad functional implications. Early occupational therapy—particularly when embedded in play and classroom activities—has been shown to improve outcomes in these domains [94].

### 4.3. Middle Childhood

Middle childhood is a period of consolidation and expansion in cognitive abilities, with formal schooling placing increasing demands on sustained attention, information processing, and problem-solving. At this stage, processing speed plays a critical role in academic success, influencing reading fluency, arithmetic fact retrieval, and classroom productivity. Survivors treated for CNS malignancies or exposed to intensive CNS-directed therapies frequently demonstrate slowed processing speed due to persistent white matter disruption and reduced neural connectivity efficiency [95,96]. This lag can make it difficult to complete tasks under time constraints, participate fully in fast-paced classroom discussions, or keep up with note-taking during lectures, leading to cumulative learning disadvantages over time.

Attention and working memory demands increase sharply during middle childhood as academic tasks require sustained focus and the ability to juggle multiple pieces of information simultaneously. Many CCSs show reduced capacity for selective and sustained attention, often coupled with weaknesses in working memory updating [97]. These challenges can impair the ability to follow multi-step instructions in math, keep track of storylines in reading, or manage multi-phase science experiments. Deficits in this domain may also affect social functioning, as children struggle to follow the flow of group play or peer conversations, potentially impacting peer acceptance [98].

Memory functions—especially declarative and episodic memory—become increasingly important for storing and retrieving large amounts of academic content. Treatment-related hippocampal injury or disruptions to fronto-temporal connectivity can hinder the encoding, consolidation, and retrieval of newly learned material [99]. In practical terms, this may manifest as difficulty recalling math procedures, remembering vocabulary definitions, or retaining information from earlier lessons when it is revisited in assessments [100]. Without targeted supports such as memory strategy training and rehearsal-based learning, these difficulties can widen achievement gaps.

Executive functions such as organization, planning, and cognitive flexibility are heavily engaged in middle childhood as academic tasks become more complex and multi-layered. Survivors may experience difficulty breaking assignments into manageable steps, adapting to new problem-solving strategies, or switching between subjects [101]. Neuroimaging studies show persistent alterations in prefrontal cortex functioning in some survivors, which may underpin these EF challenges [102]. Such deficits can have cascading effects on independent learning and homework completion, especially without structured environmental supports.

Academic achievement is a sensitive marker of neurocognitive health in this age group. Survivors may show underachievement in mathematics, reading comprehension, and written expression compared to peers [103]. Deficits in foundational domains such as processing speed and working memory often contribute to these academic struggles, leading to a gradual widening of the performance gap as curricular demands increase [104]. Without ongoing school-based accommodations, the risk of grade retention or reduced academic self-concept rises significantly.

Motor and visual–motor skills in middle childhood support tasks such as handwriting fluency, diagram copying, and coordination in sports and physical education. Treatment-related motor deficits, such as those from chemotherapy-induced peripheral neuropathy or cerebellar injury, may reduce handwriting speed, legibility, or accuracy in copying from the board [105]. These impairments can slow completion of written tasks and affect both academic and extracurricular participation. Intervention through occupational and physical therapy—particularly when integrated into the school context—can help maintain functional independence and engagement [106].

### 4.4. Adolescence

Adolescence is a critical period for the refinement of higher-order cognitive skills, increasing autonomy, and preparation for adult roles in education, employment, and social life. During this stage, processing speed is essential for coping with the pace and complexity of secondary education, where multiple subjects and high-stakes assessments demand rapid comprehension and response. Survivors of childhood cancer who experienced CNS-directed therapies often continue to show slower processing speed compared to peers [107]. This can translate into difficulty completing timed exams, keeping pace in note-taking during lectures, and efficiently integrating information from multiple sources. As secondary curricula are often less scaffolded than in earlier schooling, slow processing speed can lead to cumulative disadvantages in both academic performance and confidence.

Attention and working memory are heavily taxed in adolescence, as students must juggle competing academic, extracurricular, and social demands. Deficits in sustained attention and working memory updating can undermine study efficiency and the ability to manage multiple assignments with overlapping deadlines [108]. These weaknesses may also impact driving readiness, where attentional control and real-time information processing are crucial [109]. In social contexts, attentional lapses can impair the interpretation of nuanced social cues, potentially leading to misunderstandings or social withdrawal.

Memory demands escalate significantly in adolescence due to the volume and complexity of material in secondary education. Verbal memory deficits, particularly in delayed recall, can hinder success in subjects that require mastery of detailed information such as biology or history [110]. Adolescents with hippocampal injury or disrupted fronto-temporal pathways may require more repetitions and retrieval practice to retain material [111]. Without targeted study strategies—such as elaborative rehearsal or spaced retrieval—these survivors may underperform despite adequate understanding during initial learning.

Executive functions reach their most complex form in adolescence, enabling planning for long-term goals, prioritization of tasks, and adaptive problem-solving. Survivors may exhibit challenges in organizing workloads, anticipating deadlines, and adjusting strategies when faced with obstacles [112]. Emotional regulation, an aspect of executive functioning linked to prefrontal–limbic integration, may also be affected, increasing vulnerability to anxiety or depressive symptoms [113]. These EF difficulties can compromise readiness for post-secondary transitions, making structured vocational or academic planning support critical.

Academic achievement in adolescence reflects the cumulative impact of earlier neurocognitive deficits. Survivors may continue to experience gaps in mathematics problem-solving, reading comprehension, and writing proficiency [114]. In STEM disciplines, where conceptual understanding builds on sequential mastery of skills, these gaps can result in early disengagement from advanced coursework. Standardized test performance may be disproportionately affected, limiting access to higher education opportunities unless accommodations are secured.

Motor and visual–motor skills at this age underpin a wide range of academic and life skills, from precise laboratory work to driving. Persistent fine motor deficits can affect note-taking efficiency, while gross motor challenges may limit participation in sports or vocational activities requiring manual dexterity [115]. In some cases, these difficulties contribute to reduced social participation and physical fitness, reinforcing the importance of continued occupational and physical therapy when indicated.

## 5. Emotional Outcomes in Childhood Cancer Survivors

While neurocognitive outcomes often receive the greatest attention, emotional and psychosocial challenges are equally consequential. Anxiety, depression, and social withdrawal not only reduce quality of life but can also undermine educational and vocational attainment. Addressing these outcomes requires recognition that emotional development is inseparable from neurocognitive functioning, with both jointly shaping long-term adaptation. Emotional outcomes in childhood cancer survivors (CCS) are shaped by an interplay of neurobiological, psychological, and social factors that vary across developmental stages. Treatment-related CNS injury, prolonged medical stress, and disruptions in normative socialization can all influence emotional well-being. By examining emotional domains through a developmental lens, we can better understand when certain vulnerabilities emerge and how they evolve.

### 5.1. Infancy and Toddlerhood

In infancy, emotional development is grounded in the formation of secure attachment relationships and the regulation of physiological arousal. Survivors treated at this age may experience disruptions in emotion regulation, often related to repeated hospitalizations, separation from primary caregivers, and exposure to painful medical procedures [116]. These stressors can heighten reactivity to sensory input, making infants more prone to distress in unfamiliar or stimulating environments. Attachment patterns may also be affected, particularly when medical routines limit consistent caregiver contact. Some infants may display heightened clinginess or, conversely, reduced social engagement, both of which can signal early disruptions in socio-emotional development [117].

### 5.2. Early Childhood

By early childhood, children are developing more sophisticated ways of expressing and understanding emotions. CCSs treated during this period may exhibit heightened anxiety, particularly anticipatory anxiety related to medical contexts [118]. Cognitive immaturity at this stage limits their ability to understand the purpose of medical interventions, which can lead to fears that generalize to non-medical settings, such as preschools or social gatherings. Social-emotional competence—including sharing, empathy, and cooperative play—can be affected by reduced peer contact during treatment. Limited opportunities for peer interaction may slow the development of social skills, leading to social withdrawal or difficulties in group play. This, in turn, can exacerbate feelings of isolation and hinder socio-emotional resilience [119].

### 5.3. Middle Childhood

In middle childhood, peer relationships and academic performance become increasingly central to self-esteem. Survivors in this stage may experience elevated internalizing symptoms such as anxiety and depression, often linked to concerns about physical differences (e.g., hair loss and growth delay) and academic struggles [120]. Social integration can be particularly challenging. Persistent absences from school and difficulty keeping pace academically may lead to reduced peer acceptance, which can heighten the risk of loneliness and further emotional distress [121]. Resilience at this stage is strongly influenced by supportive peer networks, family cohesion, and school environments that actively facilitate reintegration.

### 5.4. Adolescence

Adolescence brings a heightened focus on autonomy, identity formation, and future planning. For survivors, these developmental tasks can be complicated by ongoing health concerns, cognitive late effects, and altered life trajectories. Emotional well-being in this period often reflects the cumulative effects of earlier challenges. Rates of anxiety, depression, and post-traumatic stress symptoms (PTSS) remain elevated in some survivors, particularly among those with visible treatment-related changes or ongoing functional limitations [122]. Identity development can be shaped by the cancer experience, with some adolescents integrating survivorship into a positive self-concept, while others may struggle with feelings of difference and uncertainty about the future. Concerns about fertility, long-term health, and life expectancy can also weigh heavily, influencing mood and motivation [123].

Supportive interventions during adolescence often focus on enhancing coping strategies, promoting independence while maintaining access to appropriate accommodations, and facilitating peer connections to counteract isolation. Research also highlights the intersection between executive dysfunction and emotional disturbances in childhood cancer survivors. For example, poor self-regulation, low working memory capacity, and attentional deficits can exacerbate emotional reactivity and increase susceptibility to anxiety, depression, and social withdrawal [24]. Conversely, emotional dysregulation—stemming from trauma, disrupted attachment, or prolonged hospitalizations—can undermine cognitive flexibility and planning, reinforcing a cycle of psychosocial vulnerability. A summary of cancer types, treatment modalities, and their associated neurocognitive and emotional sequelae is presented in Table 1 to provide a visual overview for clinicians and researchers.

### 5.5. Family Dynamics and Environmental Influences

Family context is a critical determinant of both neurocognitive and emotional outcomes in childhood cancer survivors, and its influence evolves across developmental stages. In infancy and toddlerhood, parents are the primary regulators of environmental stimulation and emotional security. Prolonged hospitalizations, treatment-related disruptions to daily routines, and parental distress can interfere with the establishment of secure attachment, which is foundational for stress regulation and socio-emotional development [25,26]. Studies have shown that high parental stress during early treatment predicts later difficulties in both cognitive and emotional regulation, suggesting that early psychosocial support for caregivers can have long-term benefits [27,28].

In early childhood, as children begin to expand their social world through preschool and peer play, family support continues to serve as a protective factor against treatment-related neurocognitive risks. Responsive parenting, a cognitively enriched home environment, and active engagement in play-based learning have been associated with better executive function and language outcomes in survivors [29,30]. Conversely, high levels of parental overprotection, while often motivated by concern for the child’s health, have been linked to reduced autonomy, limited peer interaction, and increased internalizing symptoms [31].

By middle childhood, survivors spend more time in school and extracurricular settings, but family engagement remains essential. Parents who maintain close collaboration with teachers, advocate for educational accommodations, and provide structured routines at home help mitigate academic and social difficulties [32,33]. Research also highlights the role of siblings in providing emotional support and facilitating social skill development, with positive sibling relationships linked to improved coping and reduced loneliness in CCS [34].

In adolescence, the family’s role shifts toward supporting autonomy, identity formation, and preparation for future roles. This developmental stage is marked by increased self-advocacy in healthcare and education, and families that encourage independence while providing a safety net foster greater resilience [35,36]. Open family communication about survivorship issues, fertility concerns, and long-term health surveillance has been associated with lower psychological distress and better quality of life in adolescent survivors [37]. Adaptive family functioning—balancing protection with autonomy—emerges as a key predictor of successful transition into adulthood for this group [38].

Cultural and contextual factors further shape survivorship experiences. Variability in caregiving practices, access to educational resources, and stigma associated with illness may amplify or buffer late effects across societies. Recent advances in digital and telehealth-based family interventions also hold promise for broadening access to psychosocial support, particularly in low-resource or geographically dispersed settings. Ultimately, children’s resilience is built within families and communities. While treatment exerts biological effects, the family environment often determines whether those effects translate into long-term impairment or adaptation. Recognizing families as active partners in survivorship care is therefore essential.

### 5.6. Methodological Considerations in Survivor Research

Research on childhood cancer survivorship faces several methodological challenges, many of which are compounded when outcomes are examined through a developmental lens. One of the foremost considerations is age at diagnosis and treatment exposure, as neurodevelopmental trajectories and environmental contexts differ markedly across infancy, early childhood, middle childhood, and adolescence [39,40]. This variability means that identical treatment protocols may yield divergent neurocognitive and emotional outcomes depending on the developmental stage at which they are administered.

In infancy and toddlerhood, assessment is complicated by the reliance on observational and caregiver-report measures, which may be influenced by parental stress or limited by the child’s emerging communication abilities [41]. Additionally, the rapid pace of developmental change in these years requires the use of sensitive, age-specific tools capable of capturing subtle delays in early sensory–motor and regulatory domains. In early childhood, methodological challenges include differentiating treatment-related effects from normative developmental variability and accounting for environmental influences such as preschool quality and peer exposure [42]. Small changes in executive function or language development at this stage can have cascading effects, yet may be overlooked in broad developmental screening tools. Middle childhood assessments benefit from greater availability of standardized academic and neuropsychological tests; however, results can be confounded by differences in school curricula, regional educational policies, and access to special education services [43]. Longitudinal studies in this age group are particularly valuable for tracking the emergence of late effects, but attrition rates can be high as families transition to less frequent medical follow-up. In adolescence, methodological issues often center on measuring complex constructs such as identity formation, autonomy, and future orientation, which require validated tools sensitive to cancer-related life course alterations [44]. Self-report becomes increasingly important, but results must be interpreted alongside objective measures and parental perspectives to capture a comprehensive picture of functioning [45].

Across all developmental stages, survivorship research is further complicated by heterogeneity in diagnosis, treatment modalities, and time since treatment completion, as well as by small sample sizes that limit statistical power [46]. Multi-site collaborations, harmonization of assessment protocols, and the integration of developmental neuroscience methods—such as neuroimaging and ecological momentary assessment—offer promising avenues for improving both the precision and comparability of findings [47,48].

## 6. Neuropsychological Assessment in Survivorship

Neuropsychological assessment is essential in understanding the impact of childhood cancer and its treatments on survivors’ cognitive and emotional functioning. A developmental approach enhances the interpretability of results by situating findings within age-appropriate expectations and trajectories.

### 6.1. Assessment Tools and Practices

Assessment practices must be adapted to developmental stage to ensure validity and sensitivity. In infancy and toddlerhood, evaluation typically relies on standardized developmental scales such as the Bayley Scales of Infant and Toddler Development or the Mullen Scales of Early Learning [49,50]. These tools capture sensory–motor integration, early language, and emerging problem-solving skills, but may not fully detect subtle neurocognitive impacts that manifest later. Observational methods and caregiver-report instruments, such as the Ages and Stages Questionnaires, are valuable complements at this stage [51].

In early childhood, formal testing can include the Wechsler Preschool and Primary Scale of Intelligence (WPPSI) alongside targeted measures of executive function (e.g., NEPSY-II subtests) and early academic readiness [52]. Play-based assessments are often effective for maintaining engagement while gathering reliable data. However, performance can be influenced by fatigue, anxiety in testing contexts, and the effects of recent medical procedures [53]. For middle childhood, standardized measures such as the Wechsler Intelligence Scale for Children (WISC-V), Wide Range Achievement Test (WRAT), and curriculum-based evaluations allow more precise identification of academic skill gaps [54]. Executive function batteries, including the Delis–Kaplan Executive Function System (D-KEFS), provide detailed profiles of cognitive flexibility, planning, and inhibitory control. Integration of school-based data, such as teacher ratings and classroom performance, is recommended to contextualize test findings [55]. In adolescence, assessments extend to higher-order cognitive skills, vocational readiness, and social-emotional functioning. The Wechsler Adult Intelligence Scale (WAIS-IV) can be introduced for older adolescents, alongside measures of metacognition, complex problem-solving, and psychosocial adjustment (e.g., Behavior Assessment System for Children—BASC-3) [56]. Computerized batteries and performance-based functional tasks can add ecological validity, reflecting real-world demands encountered during transition to adulthood [57].

### 6.2. Factors Affecting Assessment Outcomes

Multiple factors can influence neuropsychological assessment results across developmental stages. Medical variables, such as age at diagnosis, treatment modality, and presence of CNS-directed therapy, interact with neurodevelopmental processes to shape test performance [58]. Younger children may be more vulnerable to diffuse white matter injury, while older children and adolescents may experience more focal disruptions that affect specific domains [59].

Environmental factors also play a role. In infancy and early childhood, socioeconomic status, parental education, and home cognitive stimulation can mediate recovery and skill acquisition [60]. In middle childhood, access to high-quality schooling, special education resources, and peer support can influence outcomes. Adolescents may be particularly sensitive to the quality of transition planning and availability of vocational or higher education accommodations [61]. Test-related factors include fatigue, pain, and emotional state at the time of assessment, which can disproportionately affect younger children and those with ongoing medical needs [62]. Cultural and linguistic considerations are important at all ages, with evidence showing that language mismatches between assessor and participant can reduce accuracy in identifying true deficits [63]. Finally, longitudinal consistency in assessment tools is critical for tracking developmental changes over time. However, shifts in measurement tools as children age (e.g., from WPPSI to WISC to WAIS) can introduce variability unrelated to actual cognitive change [64]. Methodologically, harmonization of measures across cohorts and integration of neuroimaging or biomarker data can improve both precision and interpretability of developmental trajectories in CCS [65,66].

Feasibility considerations are important, particularly in low-resource settings where gold-standard neuropsychological tools may be unavailable. In such contexts, brief validated screening tools and teacher or caregiver ratings can provide essential insights. Emerging ecological assessments—including digital platforms, mobile applications, and wearable technologies—offer promising avenues for capturing cognitive and emotional functioning in everyday environments, complementing standardized test batteries.

In practice, this means that neuropsychological assessment should not be treated as a one-off evaluation, but as a developmental process in itself. Regular reassessment, linked to transitions such as school entry or adolescence, ensures that emerging vulnerabilities are identified and supported in real time.

## 7. Interventions and Support Strategies

Support for childhood cancer survivors (CCSs) must be developmentally tailored to optimize neurocognitive, emotional, and functional outcomes. Intervention approaches that are effective at one developmental stage may require substantial adaptation for another, reflecting differences in cognitive maturity, social roles, and environmental demands.

### 7.1. Cognitive Rehabilitation

In infancy and toddlerhood, cognitive rehabilitation often centers on enhancing sensory–motor integration, early language acquisition, and attention regulation. Interventions typically involve play-based therapy, caregiver coaching, and enriched home environments that promote active exploration [67]. Evidence shows that early intervention programs, when initiated soon after treatment, can support neural plasticity and mitigate early delays [68]. In early childhood, structured programs focusing on executive function, working memory, and language skills are critical, often integrating occupational and speech therapy [69]. For example, targeted computerized cognitive training has shown promise in improving processing speed and attention in young survivors of CNS tumors [70]. By middle childhood, school-based cognitive remediation programs become a core component of intervention, with individualized strategies addressing academic skill gaps and metacognitive awareness [71]. Computer-assisted training for working memory and problem-solving, combined with teacher-led accommodations, has been associated with sustained gains in academic performance [72]. In adolescence, cognitive rehabilitation focuses on higher-order executive skills, self-monitoring, and preparation for post-secondary education or employment. Strategies include assistive technology for organization and memory, as well as coaching in self-advocacy to secure accommodations in academic or workplace settings [73].

### 7.2. Psychosocial and Emotional Support

In infancy and toddlerhood, psychosocial support primarily involves caregiver interventions to promote secure attachment, reduce parental stress, and establish consistent routines [74]. Hospital-based family counseling and parent–infant bonding programs can buffer early emotional vulnerabilities [75]. In early childhood, interventions often address treatment-related anxiety, separation distress, and social skill development. Play therapy and group-based social skills training can help normalize experiences and facilitate peer integration [76]. In middle childhood, peer support programs, mentoring, and group counseling can address self-esteem, body image concerns, and social reintegration challenges [77]. Interventions that involve both parents and children are particularly effective in reducing internalizing symptoms and enhancing coping strategies [78]. In adolescence, therapy often focuses on identity formation, autonomy, and future planning. Cognitive–behavioral therapy (CBT), acceptance and commitment therapy (ACT), and resilience-based group programs have shown efficacy in reducing anxiety, depression, and post-traumatic stress symptoms in this population [79].

Family-based interventions that target both caregiver well-being and child outcomes appear especially impactful, reducing parental stress while improving children’s regulation and academic functioning. At a systems level, policy measures such as insurance coverage, integration of neurocognitive monitoring into national survivorship guidelines, and investment in cross-sector coordination (healthcare–education–social services) are crucial to ensure equitable access. Attention to disparities in socioeconomic status, minority background, and geographical location is also needed to reduce inequities in survivorship care.

### 7.3. Educational and School Reintegration

In infancy and early childhood, school reintegration is not yet applicable, but early educational engagement through high-quality preschool programs can support cognitive and social development [80]. Early coordination with future educators can ensure awareness of the child’s medical and developmental history [81]. In middle childhood, formal reintegration into school following treatment is a key milestone. Effective strategies include gradual re-entry, provision of individualized education plans (IEPs), and collaboration between healthcare providers, educators, and families [82]. Teacher education about cancer-related late effects is essential to reduce stigma and ensure appropriate accommodations [83]. In adolescence, school reintegration focuses on maintaining academic progress despite lingering cognitive or health challenges, as well as preparing for post-secondary education or vocational training. Transitional planning should include career counseling, assistance with standardized testing accommodations, and linkage to disability services in higher education [84].

### 7.4. Innovative Therapies and Recreational Programs

Across all developmental stages, innovative and recreational interventions play a vital role in enhancing quality of life and promoting holistic well-being. In infancy and toddlerhood, music therapy, sensory play, and aquatic programs can support sensory processing and parent–child bonding [85]. In early and middle childhood, therapeutic recreation programs such as adaptive sports, art therapy, and drama workshops provide opportunities for mastery, creativity, and social engagement [86]. Participation in medically supervised camps has been shown to improve self-confidence, peer relationships, and overall life satisfaction [87]. In adolescence, technology-based interventions—including virtual reality for stress reduction, gamified physical rehabilitation, and online peer support networks—are gaining traction [88]. These approaches can enhance motivation, improve physical activity levels, and provide a safe space for discussing survivorship challenges.

Taken together, to enhance clarity and comparability, results were summarized in Table 2, which presents key cognitive and emotional domains affected at each developmental stage, along with brief notes on study design limitations. Survivorship care is most effective when it moves beyond isolated programs to provide coordinated developmental support across healthcare, school, and family systems. The evidence makes clear that timely, context-sensitive interventions can redirect at-risk developmental pathways toward resilience and thriving.

## 8. Discussion

This review delineates how specific mechanisms (e.g., white-matter disruption after CNS-directed therapy, hippocampal injury, endocrine and ototoxic late effects) intersect with developmental timing and context (family, school) to shape outcomes. Rather than repeating section-level summaries, herein we integrate patterns across stages: (i) processing speed/working memory deficits are most evident after cranial RT or high-dose MTX, especially when exposure occurs before middle childhood (e.g., [7,31,46,49]); (ii) higher-order EF inefficiencies persist into adolescence and predict functional attainment (e.g., [6,12,17]); and (iii) internalizing symptoms (anxiety/depression/PTSS) are amplified when cognitive inefficiencies and school barriers co-occur (e.g., [17,18]). These cross-cutting regularities, not just domain checklists, should guide screening and intervention. These developmental patterns collectively suggest that early CNS-directed therapy disrupts neurodevelopmental processes at sensitive periods, with later cumulative effects on executive control and socioemotional regulation.

Associations between CNS-directed therapy and slowed processing speed/working memory are supported by prospective or cohort designs with standardized neuropsychology and, in many cases, neuroimaging correlates (e.g., [30,31,48,49]). However, heterogeneity in diagnosis/exposure and occasional absence of active control groups limit causal inference in some reports (e.g., [7,8]). Emotional outcomes are consistently elevated in large cohort analyses (e.g., CCSS, St. Jude Lifetime: [16,17,18]), but effect sizes vary with measurement approach (self- vs. parent-report) and survivorship era, underscoring the need for harmonized, longitudinal assessments. Where small samples or retrospective designs predominate (e.g., specific tumor subtypes or national subcohorts), we treat findings as hypothesis-generating rather than definitive.

Cross-sectional studies risk obscuring developmental change, and the use of age-inappropriate assessment tools can underestimate functional limitations [41,42]. Multi-modal strategies that combine standardized testing, informant reports, and neuroimaging offer more comprehensive insight [47,48], while harmonization of protocols across sites is crucial for enabling large-scale synthesis [46,102]. These approaches should also incorporate cultural and linguistic adaptations to ensure validity across populations [63].

The evidence clearly supports early, developmentally tailored interventions. In infancy and early childhood, caregiver-focused programs that promote attachment security, cognitive enrichment, and stress reduction have shown promise in reducing later difficulties [74,75,103]. Middle childhood interventions are most effective when school-based cognitive remediation is paired with social–emotional learning initiatives [71,72,104], whereas adolescent interventions should integrate vocational readiness, mental health support, and self-advocacy training [73,79,105,106]. From a policy standpoint, embedding survivorship care into national pediatric oncology protocols and ensuring cross-sector coordination between healthcare, education, and social services is essential for continuity of care [107]. Survivorship planning should extend into adulthood, bridging pediatric and adult healthcare systems, to address cumulative risks across the lifespan. From a societal perspective, late neurocognitive and emotional effects also carry significant implications for educational attainment, employment, and long-term productivity, highlighting the public health relevance of early, sustained intervention.

From a translational perspective, the present synthesis highlights several concrete priorities for clinical practice, education, and research. In clinical contexts, developmental neuropsychological screening should be integrated at key transition points—school entry, middle childhood, and adolescence—particularly for survivors exposed to CNS-directed therapy or presenting school-related or behavioral difficulties. Screening protocols should include standardized measures of processing speed, working memory, and executive functioning, alongside brief assessments of internalizing symptoms. When clinically significant deficits are identified, survivors should be promptly referred to targeted cognitive remediation and evidence-based psychosocial interventions such as cognitive-behavioral or acceptance-based therapy. Within educational systems, structured school reintegration plans should be established soon after treatment completion to ensure individualized accommodations, including reduced emphasis on speeded tasks, scaffolded learning strategies, and coordinated communication between hospitals and schools through designated liaison personnel. At a systems level, the development of survivorship care frameworks linking medical and educational data is essential for maintaining developmental continuity and preventing loss to follow-up. Finally, future research must prioritize large-scale, longitudinal, and multicenter studies with harmonized neurocognitive and emotional assessment batteries, transparent quality appraisal procedures, and explicit reporting of treatment exposures and demographic variables. Such initiatives would enable evidence-based policy, guide individualized intervention planning, and support the inclusion of neurocognitive screening as a measurable standard in pediatric oncology survivorship protocols.

Future directions for research include longitudinal studies with harmonized measures from diagnosis into adulthood [46,64], the design of multi-level interventions that address neurocognitive, emotional, and environmental factors simultaneously [97,103], and the expansion of global representation in survivorship studies to ensure culturally sensitive adaptations [63,107]. Emerging technologies such as telehealth, digital cognitive training, and virtual reality hold promise for extending reach and personalizing support [88]. Viewing survivorship through a developmental lens provides essential insights into the timing, mechanisms, and cumulative effects of late outcomes, clarifying risk periods and informing intervention timing. Integrating developmental science into clinical care, educational policy, and psychosocial support is fundamental to enabling survivors to reach their fullest potential. Ultimately, optimizing neurocognitive and emotional outcomes in childhood cancer survivors requires integrating developmental science into oncology practice, ensuring that survivorship care not only prevents late effects but also actively supports resilience, quality of life, and lifelong participation in society.

The evidence is unequivocal: neurocognitive and emotional late effects are not rare complications but core outcomes of childhood cancer survivorship. When these challenges are left unaddressed, they compromise educational progress, limit employment opportunities, and reverberate across families and communities. Conversely, when developmental science informs survivorship care—through timely assessment, family-centered intervention, and equitable access to services—children not only survive but thrive. The responsibility to act is therefore both clinical and societal: survivorship care must evolve from preventing mortality to fostering resilience, participation, and lifelong quality of life.

## Figures and Tables

**Table 1:** Summary of common cancer types, treatment modalities, and associated neuropsychological sequelae.

Cancer Type	Treatment Modalities	Cognitive Sequelae	Emotional Sequelae
ALL (Acute Lymphoblastic Leukemia)	Chemotherapy, intrathecal methotrexate, occasional cranial irradiation (historic)	Processing speed, attention, executive dysfunction, memory deficits; higher vulnerability when treated at younger ages [23,69,78]	Anxiety, depression, social withdrawal, PTSD symptoms [20,62,80]
CNS Tumors	Surgery, cranial/spinal irradiation, chemotherapy	Global IQ decline, impaired processing speed, executive dysfunction, working memory deficits; radiation dose–response effects [67,70,71,75]	Emotional dysregulation, chronic anxiety/depression, social functioning difficulties [63,73,80]
Neuroblastoma	Intensive chemotherapy, surgery, stem cell transplant, immunotherapy	Visual-motor deficits, attention and executive impairments, lower academic outcomes, especially in younger survivors [65,68,72]	Anxiety, sleep disturbances, social difficulties, treatment-related trauma responses [64,79,83]
Bone Tumors (e.g., Osteosarcoma, Ewing Sarcoma)	Surgery (amputation/limb salvage), chemotherapy, sometimes radiotherapy	Mostly preserved cognition, though chemotherapy-related attention/processing vulnerabilities possible [74,89,94]	Body image concerns, depression, anxiety, adjustment difficulties [41,73,85]
Renal Tumors (e.g., Wilms’ Tumor)	Nephrectomy, chemotherapy, occasional radiotherapy	Generally minimal late cognitive effects; subtle learning/attention difficulties in subgroups exposed to radiation or anthracyclines [62,86,90]	Anxiety about health/recurrence, internalizing symptoms; parental stress impacting adjustment [40,80,88]

Note: ALL, acute lymphoblastic leukemia; CNS, central nervous system.

**Table 2:** Summary of key neurocognitive and emotional outcomes across developmental stages and methodological notes.

Developmental Stage	Primary Affected Cognitive Domains	Emotional/Psychosocial Outcomes	Representative Studies	Main Methodological Limitations
Infancy/Toddlerhood	Delays in processing speed and emerging executive functions; early white-matter and hippocampal disruption.	Dysregulated affect; attachment insecurity; caregiver stress and anxiety.	[25,26,27,28,29,33,46,48,77,82,116]	Very small single-center samples; frequent absence of healthy control groups; cross-sectional designs; retrospective reporting; survivors treated under older protocols.
Early Childhood	Working-memory, attention, and language inefficiencies; reduced processing speed following chemotherapy or RT.	Separation anxiety; social withdrawal; early peer avoidance; fear generalization.	[8,9,10,11,12,13,14,15,16,17,18,19,20,21,22,23,24,25,26,27,28,29,30,31,32,33,34,35,36,37,38,39,40,41,42,43,44,45,46,47,48,49,50,51,52,53,54,55,56,57,58,59,60,61,62,63,64,65,66,67,68,69,70,71,72,73,74,75,76,77,78,79,80,81,82,83,84,85,86,88,118]	Lack of randomized controls; moderate sample sizes (20–80 participants); short follow-up; attrition bias; heterogeneity in imaging and testing protocols.
Middle Childhood	Slowed processing speed; deficits in executive planning, working memory, and academic achievement.	Internalizing symptoms, social isolation, reduced self-esteem.	[36,37,38,39,56,57,58,59,60,61,95,96,97,120]	Cognitive outcomes often secondary endpoints; incomplete follow-up; no healthy controls; reliance on self-report in some cohorts; heterogeneity in treatment exposures.
Adolescence	Persistent inefficiencies in higher-order executive function, working memory, and metacognition; slowed processing speed.	Depression, anxiety, post-traumatic stress symptoms, and identity difficulties during school and transition to adulthood.	[107,112,122]	Cross-sectional or retrospective designs; selective participation of higher-functioning survivors; reliance on self-report; older treatment eras; limited cultural generalizability.

Note: RT = radiation therapy. Reference numbers correspond to those cited in the Results section. The table integrates only studies already discussed in the text to maintain alignment with the narrative synthesis.

## Data Availability

No new data were created or analyzed in this study. Data sharing is not applicable to this article.

## Abbreviations

The following abbreviations are used in this manuscript:

EF	Executive Functions
ALL	Acute Lymphoblastic Leukemia
CNS	Central Nervous System
SPS	Sensory Processing Sensitivity
CBT	Cognitive–Behavioral Therapy
RCT	Randomized Controlled Trial
